# Evidence of a care home effect on antibiotic prescribing for those that transition into a care home: a national data linkage study

**DOI:** 10.1017/S0950268818003382

**Published:** 2019-04-08

**Authors:** L. Patterson, A. Maguire, C. Cardwell, F. Kee, C. Hughes, L. Geoghegan, L. Doherty, M. Dolan, N. Q. Verlander, D. O'Reilly

**Affiliations:** 1 Field Epidemiology Training Programme, Public Health England, Belfast, Northern Ireland; 2 UKCRC Centre of Excellence for Public Health (Northern Ireland), Queen's University Belfast, BT12 6BJ, Northern Ireland; 3 Public Health Agency, Health Protection, 12-22 Linenhall Street, Belfast, BT2 8BS, Northern Ireland; 4 Centre for Public Health, Queen's University Belfast, Belfast, BT12 6BJ, Northern Ireland; 5 School of Pharmacy, Queen's University Belfast, 97 Lisburn Road, Belfast, BT9 7BL, Northern Ireland; 6 The Regulation and Quality Improvement Authority, 9th Floor Riverside Tower, 5 Lanyon Place, Belfast, BT1 3BT, Northern Ireland; 7 Health and Social Care Board, 12-22 Linenhall Street, Belfast, BT2 8BS, Northern Ireland; 8 Statistics, Modelling and Economics Department, National Infection Service, Public Health England, London, UK

**Keywords:** Analysis of data, antibiotic resistance, antibiotics, epidemiology, infectious disease epidemiology

## Abstract

We compared antibiotic prescribing to older people in different settings to inform antibiotic stewardship interventions. We used data linkage to stratify individuals aged 65 years and over in Northern Ireland, 1st January 2012–31st December 2013, by residence: community dwelling, care home dwelling or ‘transitioned’ if admitted to a care home. The odds of being prescribed an antibiotic by residence were analysed using logistic regression, adjusting for patient demographics and selected medication use (proxy for co-morbidities). Trends in monthly antibiotic prescribing were examined in the 6 months pre- and post-admission to the care home. The odds of being prescribed at least one antibiotic were twofold higher in care homes compared with community dwellers (adjusted odds ratio 2.05, 95% CI 1.93–2.17). There was a proportionate increase of 51.5% in the percentage prescribed an antibiotic on admission, with a monthly average of 23% receiving an antibiotic in the 6 months post admission. While clinical need likely accounts for some of the observed antibiotic prescribing in care homes we cannot rule out more liberal prescribing, given the twofold difference between care home residents and their community dwelling peers having accounted for co-morbidities. The appropriateness of antibiotic prescribing in the care home setting should be examined.

## Introduction

Recent estimates indicate that if trends in antimicrobial resistance (AMR) continue unabated, by 2050, 10 million lives will be lost each year to AMR at a cumulative cost to global economic output of $100 trillion US dollars [1]. One of the most important drivers for the emergence of resistance is the overuse of antibiotics [2]. In the UK, most prescribing is carried out by general practitioners (GPs), and their decision to prescribe may be influenced by a range of non-clinical factors including patient demands, the physician's characteristics and attitudes and wider healthcare system factors such as culture and cost considerations [3–5].

Rates of antibiotic prescribing are highest at the extremes of age [6–8]. While interventions such as efforts to reduce inappropriate prescribing and national vaccination campaigns have helped to improve prescribing rates in the younger age groups antibiotic prescribing for the older population appears to be increasing [9]. The older population (conventionally considered to be those 65 years and older) are of particular interest as they represent an increasing proportion of our society with complex healthcare needs. Antibiotic prescribing for older people in care homes has been recognised as a global problem which raises the concern about how this may drive resistance and facilitate spread through inter-person transmission [10]. Obtaining estimates of the burden of AMR in the care home setting is challenging due to a lack of routine surveillance in this setting but a recent population based study comparing residents of long-term care facilities to their community dwelling peers showed higher levels of AMR in the care home setting [11].

For older people living in the community, the typical care pathway leading to the prescription of an antibiotic is that the patient will be assessed by a GP before a decision is made to prescribe an antibiotic. Best practice is a face-to-face consultation although GPs may choose to prescribe without direct contact on the basis of a telephone consultation or laboratory results. In the care home setting, it is generally a healthcare professional from the care home who makes contact with the GP on behalf of the resident. Preliminary clinical information may be gathered by GP administrative staff, the level of which will depend on local practice protocols. This may include some diagnostic information such as temperature, symptoms or results of urine dipstick tests. A GP in the practice will review this information and make a decision on whether to make a home visit, contact the care home for further information or issue a prescription without further contact. In the care home setting multiple GPs and practices may service the same care home with varying protocols in use. It is conceivable that the different routes to receiving a prescription in the community *vs.* in a care home might influence prescribing trends observed.

The primary objectives of this study were (i) to confirm if there are higher levels of antibiotic prescribing among residents of care homes compared with community dwelling peers and (ii) to examine antibiotic prescribing changes over time, before and after care home admission.

## Methods

This was a population-based data-linkage study of all individuals alive and resident in Northern Ireland (NI) aged 65 years and over (at the start of the study) who were followed up for a period of 24 months from 1st January 2012–31st December 2013. The cohort was defined using the demographic information within the national, central health card registration system (which all citizens must register with to obtain free access to health care), with further information on care homes and prescribing derived from linkage to the Regulation and Quality Improvement Authority (RQIA – regulatory body for care homes in NI) and the Enhanced Prescribing Database (EPD) respectively (described below).

The health card registration system holds each patient's unique identifier, the health and care number (HCN), that can be used to link health-related datasets and information on vital events such as deaths as well as an identifier for the individuals registered GP practice. Patient address information is also held and is linked electronically to GP practice computers and changed automatically when addresses are updated at the practice, such as when a patient moves to a care home [12]. Address changes for care home residences are likely to be updated quickly as GPs are notified about their patients upon permanent entry to care home to ensure provision of medications. We used deterministic linkage methods to link the patient's address information in the registration system to the official list of homes held and maintained by the RQIA using a unique property reference number, which is a unique identifier for every addressable location in the UK. All care homes in NI were included. We identified individuals in a care home at the start of the study and those admitted to a care home during the 24 months of follow-up. A similar method has been used to examine psychotropic medications and transition into care [12].

The EPD contains information about all medications prescribed by a GP and dispensed by community pharmacies in NI [12, 13]. Patients in NI are not required to pay for prescribed medications. The completeness and validity of the data are known to be excellent [14]. The EPD and health card registration system were linked using the patient's HCN. The British National Formulary (BNF) codes which contain details for all medicines that are generally prescribed in the UK were used to extract prescribing data.

The linked dataset contained the following defined at the start of the study period: age (grouped in 10 year age bands starting at 65 years), sex, an anonymised GP identifier and an indicator for residence in a care home at the start of the study (Y/N). For those admitted to a care home during the study period, month of admission was identified, chosen to be broad enough to account for potential lag times between moving into the care home and notifying the GP practice. The care homes were assigned an anonymised identifier. Using this information we created a residence type which categorised the cohort into community dwelling, transitioned (for those admitted to a care home) or care home dwelling for those who were resident from the start of the study. Month of death and monthly indicators of antibiotics dispensed (BNF 5.1, Y/N) were captured for all individuals during the 24 months. Antibiotics dispensed were also recorded for 6 months prior to the study period and 6 months after so that individuals who moved into care homes in January 2012 or December 2013 could be included in the longitudinal analysis of prescribing trends for those admitted to a care home.

To control for potential co-morbidities, the presence of a condition (Y/N) was determined using the prescription of one or more of the following medications during 2012 [15, 16]: prescriptions dispensed for cardiovascular (BNF 2), anti-diabetic (BNF 6.1), anti-inflammatory (BNF 10.1), psychotropic (BNF 4.1/4.2/4.11), respiratory (BNF 3.1–3.3) and catheters. All data were provided by the Business Services Organisation (see Table S1 for further detail about inclusions).

Ethical approval was obtained from the Office for Research Ethics Committees NI (REC reference 15/NI/0119) and the linked dataset was stored within the Honest Broker Service.

### Analytical strategy

To assess if antibiotic prescribing was higher in individuals in care homes, compared with their community dwelling peers, we compared three groups of patients who were alive in January 2012 and January 2013 (*n* = 256 763 people): community dwellers; those dwelling in care homes and those who moved from the community into a care home. Sensitivity analysis was conducted in June to allow for seasonal variation (data not shown but available on request). We compared using odds ratios (ORs) and 95% confidence intervals (CIs), with statistical significance assessed using McNemar's test.

To assess if antibiotic prescribing differed by residence type, adjusted for potential comorbidities, we analysed a closed cohort of individuals aged 65 years and over who were alive during 2012 (*n* = 257 950). Univariate logistic regression analyses tested the association between residential status, the *a priori* confounders (age and sex) and the covariates (co-morbidities) and the outcome. All variables from the univariate analysis were included in the final multivariable model (*n* = 223 145; excludes individuals with missing data (13%; individuals with missing data were less likely to be older, female and to reside in care homes, see Table S2)). For context, we also compared the demographic and co-morbidity covariates in community dwellers *vs.* those in a care home which showed that care home residents were older and more likely to be female and more likely to receive diabetic and psychiatric medication and catheters (see Table S3).

The assumption of independence between individuals’ registered with a GP practice or care home was tested on community and care home residents respectively using a multi-level logistic regression model. As the variance coefficients for both analyses were small (0.06 and 0.24 for the community and care home model respectively), we accepted the assumption of statistical independence and presented the fixed effects logistic regression (described above).

To examine changes in antibiotic prescribing on entry to the care home we analysed 6042 individuals who were admitted to a care home during the 24 months. The percentage prescribed 6 months before and after care home admission was plotted (excludes those that died within 6 months of admission (*n* = 890)). This method allowed each individual to act as their own control, controlling for confounders that wouldn't change dramatically over a 12 month period including age and sex. The month of admission was defined as month zero. The average percentage prescribed pre and post admission was calculated and 95% CIs added around this estimate using a multi-level logistic regression model with the identity link.

All supplementary material is available on the Cambridge Core website.

## Results

In January 2012, 269 891 people aged 65 years and older were registered with a GP practice in NI; 56% were female, 3.7% were residents in a care home at the outset, 2.2% transitioned into a care home during the 24 month follow-up and 9.9% died during the 24 months of follow-up. The analysis captured the movement of people into 350 care homes which were serviced by 353 practices.

In January 2012, the proportion who had been prescribed at least one antibiotic was twofold higher in individuals who were in a care home (21%) compared with those who were residents in the community (10%, Table 1), and this difference was still evident one year later in January 2013. For those in the transition category, levels of antibiotic use at baseline was similar to community dwellers at baseline (14.5% and 10.2% respectively) but at the second point was more similar to that of care home residents (26.3% and 25.3% respectively). The largest change in antibiotic use was therefore observed in those who transitioned into care, with an absolute difference between January 2012 and January 2013 of 11.8% (95% CI 9.8–13.9), and a relative change of 2.4 (OR 2.40, 95% CI 2.05–2.83). The sensitivity analyses comparing June 2012 and June 2013 produced similar but slightly attenuated results (available on request).

**Table 1.** The number and proportion of individuals aged 65 years and over prescribed one or more antibiotic according to residential status^a^ in Northern Ireland, January 2012 and January 2013

	Total population	Any antibiotic prescribed January 2012 (%)	Any antibiotic prescribed January 2013 (%)	Absolute difference (95% CI)	OR (95% CI)	*P* value
Community at both time points	247 036	25 273 (10.2)	27 517 (11.1)	0.91 (0.75, 1.07)	1.12 (1.10, 1.14)	<0.001
Transition from community to care home	2549	369 (14.5)	671 (26.3)	11.8 (9.8, 13.9)	2.40 (2.05, 2.83)	<0.001
Care home at both time points	7178	1538 (21.4)	1814 (25.3)	3.85 (2.60, 5.09)	1.31 (1.20, 1.44)	<0.001

a Excludes those that died during January 2012–January 2013.

During 2012, over 70% of those who were residents in a care home or who transitioned from the community received at least one antibiotic compared with 49% of those who resided in the community (Table 2). After adjusting for age, sex and co-morbidities, the odds of being prescribed at least one antibiotic was twofold higher for care home residents (adjusted OR (AOR) 2.05, 95% CI 1.93–2.17; Table 2). The odds of being prescribed at least one antibiotic increased with age, with a greater odds of receipt of antibiotic prescription for those aged 85+ years AOR (AOR 1.29, 95% CI 1.26–1.33) compared with those aged 65–74 years. The odds of being prescribed at least one antibiotic were 1.51 times higher in females compared with males (AOR 1.51, 95% CI 1.48–1.53). The adjusted estimates showed a 25%–50% increased odds of being prescribed an antibiotic for those prescribed cardiovascular, diabetes, inflammatory and psychiatric medication (Table 2). Further, there was almost a fourfold increased odds of being prescribed at least one antibiotic for those on respiratory medication (AOR 3.71, 95% CI 3.62–3.80; Table 2). Finally, there was a sixfold increased odds of being prescribed at least one antibiotic for those prescribed a catheter (AOR 5.99, 95% CI 5.28–6.79; Table 2).

**Table 2.** The odds of being prescribed an antibiotic for individuals aged 65 years and over, by demographic factor, residence type and individual co-morbidities in Northern Ireland, January–December 2012^a^

	*n*	Prescribed at least one antibiotic during 2012 *n* (%)	OR	95% CI	AOR^b^,^c^	95% CI
Total population	257 950	129 616 (50.3)				
Age						
65–74	146 970	68 794 (46.8)	1.00		1.00	
75–84	83 568	44 758 (53.6)	1.31	1.29–1.33	1.16	1.14–1.19
85+	27 412	16 064 (58.6)	1.61	1.57–1.65	1.29	1.26–1.33
Sex						
Male	113 236	49 758 (43.9)			1.00	
Female	144 714	79 858 (55.2)	1.57	1.55–1.60	1.51	1.48–1.53
Residence						
Community	248 125	122 364 (49.3)	1.00		1.00	
Transition	2429	1758 (72.4)	2.69	2.46–2.94	1.81	1.64–1.99
Care home	7396	5494 (74.3)	2.97	2.82–3.13	2.05	1.93–2.17
Cardiovascular medication^b^						
No	23 298	12 886 (55.3)	1.00		1.00	
Yes	199 847	107 203 (53.6)	0.93	0.91–0.96	1.27	1.24–1.31
Diabetic medication^b^						
No	190 937	101 342 (53.1)	1.00		1.00	
Yes	32 208	18 747 (58.2)	1.23	1.20–1.26	1.33	1.30–1.37
Inflammatory medication^b^						
No	174 770	92 694 (53.0)	1.00		1.00	
Yes	48 375	27 395 (56.6)	1.16	1.13–1.18	1.36	1.33–1.39
Psychiatric medication^b^						
No	157 843	78 507 (49.7)	1.00		1.00	
Yes	65 302	41 582 (63.7)	1.77	1.74–1.81	1.56	1.53–1.59
Respiratory medication^b^						
No	174 210	82 952 (47.6)	1.00		1.00	
Yes	48 935	37 137 (75.9)	3.46	3.39–3.54	3.71	3.62–3.80
Catheter^b^						
No	220 839	118 081 (53.5)	1.00		1.00	
Yes	2306	2008 (87.1)	5.86	5.19–6.62	5.99	5.28–6.79

a Excludes those that died.

b 223 145 individuals available for the analysis.

c Adjusted for all other factors in the model.

### Trends in prescribing for those that transition into care

**Fig. 1.**
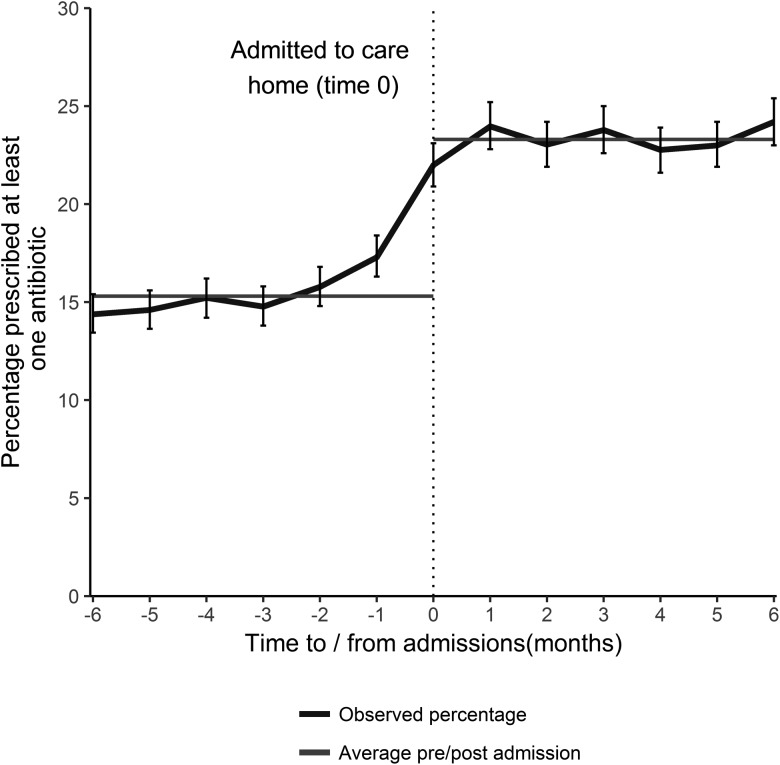
The percentage of individuals prescribed an antibiotic by time of admission (with 95% CI), and the average percentage prescribed in the 6 months prior to- and post- admission, for all those that transitioned from the community into a care home during January 2012–December 2013 (excludes those that died).

Antibiotic prescribing was relatively uniform in the 5 months prior to care home admission at approximately 15.3% (95% CI 14.9–15.7) per month. The percentage prescribed an antibiotic increased sharply in the month preceding and the month following admission to a care home (Fig. 1). The average percentage prescribed an antibiotic following admission was 23.3% (95% CI 22.8–23.6) for the first 6 months, a proportionate increase of 51.5% (absolute increase 7.9%). For those who had not received an antibiotic in the 9 months prior to care home admission (2643 individuals), 15% received an antibiotic on admission.

## Discussion

This study has shown that the odds of being prescribed at least one antibiotic are at least twice as high in care homes as they are in the community, accounting for co-morbidities. While this may be expected, given the frailty of the care home population, we believe that this is not solely due to a continuation of antibiotic prescribing to sick individuals admitted from the community. This is evidenced by the change in the percentage prescribed an antibiotic on admission to the care home and the continued high percentage that receive an antibiotic after admission. We also found that the odds of being prescribed antibiotics were 50% higher in females, compared with males which is consistent with a recent systematic review and meta-analysis in the community setting [17]. We hypothesise that this may arise for a number of reasons including differences in urinary tract infection prevalence in men and women, as well as differences in health seeking behaviour, with females residing in the community more likely to consult their GP [18].

The strength of this study included the population-wide coverage for prescribed medications, no response bias or loss to follow-up and favourable logistics (fast and inexpensive to conduct). In order to compare the percentage prescribed an antibiotic over time relative to care home admission we described antibiotic use for individuals over time, so each individual acted as their own control, which will account for background morbidity as well as age and sex. However, this does not account for acute changes to the patients’ health which may occur over the study period. We were able to identify co-morbidities by inferring disease by the prescription of particular medications. While this has not been validated for the UK population there is evidence from elsewhere that this approach is effective [15, 16]. However, disease cannot always be assumed by the treatments individuals receive and may not be a good indicator for overall frailty and so these are limitations. Another limitation of the co-morbidity data was that exposure was measured as any prescription (yes/no) during a 12 month period rather than being treated as a time-varying covariate. This was appropriate for controlling for the confounding effect of the conditions but using time-varying estimates may have improved the sensitivity of the model to accurately measure individual frailty. The choice of co-morbid conditions will also have minimised the impact of this as it is likely that in this age group individuals will have been managing their condition for some time. Further, if the identified conditions caused a sudden deterioration in health prior to their care home admission then this would have been accurately captured. We believe the results are generalisable to other populations where prescriptions are free to older people.

This study focused on the monthly period prevalence of being prescribed an antibiotic. This may have created uncertainty about the timing of the change in the percentage prescribed an antibiotic which may contribute to the slight increase in the percentage prescribed in the month preceding admission. However, more complex data would be required to fully understand this observation. We also did not capture individuals who were admitted for respite care or as part of step-down care from the acute setting and who will ultimately reside in the community. It is likely that these only account for a small proportion of care admissions and that the majority will remain in the care home once admitted and will thus be detected as a care home resident in this study [19].

The main limitations relate to interpretation and the implication. Firstly, we expect differences in antibiotic prescribing patterns when comparing community dwelling older adults to care home residents simply because the latter are likely to be sicker and have a greater clinical need. However, our analysis showed that even accounting for co-morbidities, care home residents had twofold higher odds of receiving an antibiotic compared with their community dwelling peers. To investigate the care home cohort further we described changes in prescribing pre and post care home admission for those that transitioned into a care home during the study. While this showed an absolute increase of 8% in the percentage receiving an antibiotic, comparing pre- and post-admission, the lack of clinical data means that we could not account for changes in individual frailty and thus the analysis is inconclusive. It is also possible the observed trends may be explained by an increased transmission of infection among vulnerable individuals. This is plausible as nursing homes have been shown to play a role in facilitating sustained transmission of pathogens [20].

A third explanation is that at least some of the prescribing is driven by an excess use of antibiotics. A recent systematic review suggested that almost half of prescribing to nursing home residents was potentially inappropriate [21], almost twice that of a previous estimate in a community dwelling older adult population [22]. Other studies have alluded to the common use of antibiotics in care homes [23, 24] and that therapy is often more prolonged than required [23]. This scenario is conceivable in NI care homes where the proportion of prescriptions given prophylactically is the highest in Europe [25]. Assessing the appropriateness of antibiotic prescribing in care homes requires further investigation.

There has been significant focus on hospitals in recognition of the overuse of antibiotics and the risk of antimicrobial resistant infections in this setting. However, the same concerns are evident in care homes [23]. It seems futile, therefore, to understand antibiotic use and implement control measures in one setting without complementary methods in the other. While this study identified a care home effect, it is still not known what factors are influencing the trends observed. This should be explored in the local context and may include medication reviews for appropriateness, review of treatment guidelines and catheter management [5, 25].

Consistent with the objectives outlined by the WHO Global Action plan on AMR [26], it seems another key intervention will be to improve awareness and understanding of AMR and the role of antibiotic prescribing in the care home setting. To do this, it is necessary to identify those who have a role in the care pathway for older people in care homes. GPs have a role in issuing the prescription and assuming that in most cases a GP continues to care for a patient when they are admitted to a care home [27] they are an important group. Acknowledging the role of the public, in this case the families and the individuals themselves, another key group are the care home staff. These staff members serve as a conduit between care home patients and their GPs and it could be some aspect of this communication that influences the trends observed. Therefore, education about antibiotic prescribing and AMR should not be limited to staff members and local prescribers but should be extended to the residents and their family members so that AMR becomes everyone's responsibility [5, 28]. While conventional education may be necessary, it is likely not to be sufficient for change and whole system based and ‘nudge’ approaches should be considered [29].

## Supplementary material

For supplementary material accompanying this paper visit https://doi.org/10.1017/S0950268818003382. click here to view supplementary material
