# Supplementary material for: Evidence of a care home effect on antibiotic prescribing for those that transition into a care home: a national data linkage study
Source: Epidemiol Infect. 2019 Apr 8;147:e115. doi: 10.1017/S0950268818003382 (PMC6518826; doi:10.1017/S0950268818003382)
Supplement: Supplementary file 1 [file S0950268818003382sup001.docx]

*Epidemiology and Infection*

Title: Evidence of a care home effect on antibiotic prescribing for those that transition into a care home: a national data linkage study.

Authors: L. Patterson, A. Maguire, C. Cardwell, F. Kee, C. Hughes, L. Geoghegan, L. Doherty, M. Dolan, N. Q. Verlander, D. O’Reilly

Supplementary material

Supplementary Table S1 BNF categories for co-variates included in the analysis

| **BNF Chapter** | **BNF Section** | **BNF code (chapter.section)** |
| --- | --- | --- |
| ***Appliances^[[1]](#footnote-1)^*** | *Catheters* | *21.2* |
| **Cardiovascular System** | Anti-Arrhythmic Drugs | 2.3 |
|  | Anticoagulants & Protamine | 2.8 |
|  | Antifibrinolytic Drugs & Haemostatics | 2.11 |
|  | Antiplatelet Drugs | 2.9 |
|  | Beta-Adrenoceptor Blocking Drugs | 2.4 |
|  | Diuretics | 2.2 |
|  | Hypertension & Heart Failure | 2.5 |
|  | Lipid-Regulating Drugs | 2.12 |
|  | Nitrates,Calcium-Channel.Blockers & Other Antianginal Drugs | 2.6 |
|  | Positive Inotropic Drugs | 2.1 |
|  | Sympathomimetics | 2.7 |
| **Central Nervous System** | Drugs For Dementia | 4.11 |
|  | Drugs Used In Psychoses & Related Disorders | 4.2 |
|  | Hypnotics & Anxiolytics | 4.1 |
| **Endocrine System** | Drugs Used In Diabetes | 6.1 |
| **Musculoskeletal & Joint Diseases** | Drugs Used In Rheumatic Diseases & Gout | 10.1 |
| **Respiratory System** | Bronchodilators | 3.1 |
|  | Corticosteroids | 3.2 |
|  | Cromoglycate & Related Therapy, Leukotriene  Receptor Antagonists | 3.3 |

Supplementary Table S2 Analysis of individuals aged 65 years and over with missing data, January – December 2012.

|  | **Missing data** | |  |  |  |  |
| --- | --- | --- | --- | --- | --- | --- |
|  | **Yes (%)** | **No (%)** | **OR** | **95% CI** | **AOR*** | **95% CI** |
| **Total population (n=255521)** |  |  |  |  |  |  |
| **Age** |  |  |  |  |  |  |
| 65-74 | 24,814 (16.9) | 122,156 (83.1) | 1 |  |  |  |
| 75-84 | 7,813 (9.35) | 75,755 (90.7) | 0.51 | 0.49, 0.52 | 0.52 | 0.51, 0.54 |
| 85+ | 2,178 (7.95) | 25,234 (92.1) | 0.42 | 0.41, 0.44 | 0.48 | 0.46, 0.50 |
| **Sex** |  |  |  |  |  |  |
| Male | 16,679 (14.7) | 95, 557 (85.3) | 1 |  |  |  |
| Female | 18,126 (12.5) | 126,588 (87.5) | 0.83 | 0.81, 0.85 | 0.89 | 0.87, 0.91 |
| **Residence in 2012** |  |  |  |  |  |  |
| Community | 34,357 (13.9) | 213,768 (86.2) | 1 |  |  |  |
| Transitioned | 67 (2.8) | 2,362 (97.2) | 0.18 | 0.14, 0.23 | 0.25 | 0.20, 0.32 |
| Care home | 381 (5.2) | 7,015 (94.9) | 0.34 | 0.30, 0.37 | 0.48 | 0.44, 0.54 |

*adjusted for all other factors in the model

Supplementary Table S3 A comparison of age, sex and co-morbidities for care home and community dwelling older people in Northern Ireland, January – December 2012.*

|  | **Care home (%)** | **Community (%)** | **OR** | **95% CI** | **AOR^1,ǂ^** | **95% CI** |
| --- | --- | --- | --- | --- | --- | --- |
| **Total population (n=255521)** | **7,396** | **248,125** |  |  |  |  |
| **Age** |  |  |  |  |  |  |
| 65-74 | 975 (13.2) | 145,667 (58.7) | 1 |  |  |  |
| 75-84 | 2,725 (36.8) | 79,886 (32.2) | 5.1 | 4.73, 5.49 | 4.04 | 3.74, 4.37 |
| 85+ | 3,696 (50.0) | 22,572 (9.1) | 24.5 | 22.8, 26.3 | 16.1 | 14.9, 17.3 |
| **Sex** |  |  |  |  |  |  |
| Male | 1,953 (26.4) | 110,524 (44.5) | 1 |  |  |  |
| Female | 5,443 (73.6) | 137,601 (55.5) | 2.24 | 2.12, 2.36 | 1.41 | 1.33, 1.49 |
| **Cardiovascular meds** (n=220783) |  |  |  |  |  |  |
| No | 1,043 (14.9) | 22,010 (10.3) | 1 |  |  |  |
| Yes | 5,972 (85.1) | 191,758 (89.7) | 0.66 | 0.61, 0.70 | 0.62 | 0.57, 0.67 |
| **Diabetic meds** (n=220783) |  |  |  |  |  |  |
| No | 5,918 (84.4) | 182,991 (85.6) | 1 |  |  |  |
| Yes | 1,097 (15.6) | 30,777 (14.4) | 1.1 | 1.03, 1.18 | 1.45 | 1.35, 1.56 |
| **Inflammatory meds**  (n=220783) |  |  |  |  |  |  |
| No | 6,566 (93.6) | 166,070 (77.7) | 1 |  |  |  |
| Yes | 449 (6.40) | 47,698 (22.3) | 0.24 | 0.22, 0.26 | 0.33 | 0.30, 0.37 |
| **Psychiatric meds** (n=220783) |  |  |  |  |  |  |
| No | 1,886 (26.9) | 155,331 (72.7) | 1 |  |  |  |
| Yes | 5,129 (73.1) | 58,437 (27.3) | 7.23 | 6.85, 7.63 | 5.42 | 5.12, 5.74 |
| **Respiratory meds** (n=220783) |  |  |  |  |  |  |
| No | 5,690 (81.1) | 166,587 (77.9) | 1 |  |  |  |
| Yes | 1,325 (18.9) | 47,181 (22.1) | 0.82 | 0.77, 0.87 | 0.87 | 0.82, 0.93 |
| **Catheter** (n=220783) |  |  |  |  |  |  |
| No | 6,658 (94.9) | 211,950 (99.2) | 1 |  |  |  |
| Yes | 357 (5.09) | 1,818 (0.85) | 6.25 | 5.57, 7.02 | 4.37 | 3.82, 4.99 |

*excludes those that died

^1^Final model includes 220,783 individuals

ǂadjusted for all other factors in the model

1. *NHS Prescription Services have created pseudo BNF chapters for items not included in BNF chapters 1 to 15. The majority of such items are dressings and appliances, which have been classified into four pseudo BNF chapters (20 to 23). Chapters 18 and 19 include preparations used in diagnosis, and ‘other preparations’. Sources:* [*http://content.digital.nhs.uk/media/10686/Download-glossary-of-terms-for-GP-prescribing---presentation-level/pdf/PLP_Presentation_Level_Glossary_April_2015.pdf*](http://content.digital.nhs.uk/media/10686/Download-glossary-of-terms-for-GP-prescribing---presentation-level/pdf/PLP_Presentation_Level_Glossary_April_2015.pdf) [↑](#footnote-ref-1)
